# Quantitative Evaluation of the Effect of Temperature on Magnetic Barkhausen Noise

**DOI:** 10.3390/s21030898

**Published:** 2021-01-29

**Authors:** Yujue Wang, Turgut Meydan, Yevgen Melikhov

**Affiliations:** Wolfson Centre for Magnetics, School of Engineering, Cardiff University, Cardiff CF24 3AA, UK; wangy252@cardiff.ac.uk (Y.W.); melikhov@cardiff.ac.uk (Y.M.)

**Keywords:** magnetic Barkhausen noise, temperature, thermal stress, nondestructive evaluation

## Abstract

The effect of temperature on magnetic Barkhausen noise (MBN) can be divided into two types: the direct effect of temperature itself and the indirect effect of thermally induced stress. The theoretical model is proposed in this paper to describe the effects of temperature on the MBN signal. For the case considering the direct effect of temperature only, the analytical model allows the prediction of the effect of temperature on MBN profile, and, based on the model, a simple linear calibration curve is presented to evaluate the effect of temperature on MBN amplitude quantitatively. While for the case where the indirect effect of thermal stress is taken into account in addition to the direct effect, the proposed theoretical model allows the deduction of parabolic function for quantitative evaluation of the combined effect on MBN. Both effects of temperature on MBN, i.e., the direct only and the combined one, have been studied experimentally on 0.5 mm thickness non-oriented (NO) electrical steel and the adhesive structure of NO steel and ceramic glass, respectively. The reciprocal of the measured MBN peak amplitude (1/*MBN_p_*) in the first case shows a linear function of temperature, which agrees with the proposed linear calibration curve. While in the experiments considering the combined effects, 1/*MBN_p_* shows parabolic dependence on temperature, which is further simplified as a piecewise function for the practical applications.

## 1. Introduction

The magnetic Barkhausen noise (MBN) is generated by the discontinuous domain wall motion and domain transition in the ferromagnetic materials subjected to a changing magnetic field [1]. During these processes, pinning sites, local microstructural defects, and stresses (local and global) jointly contribute to the discontinuous stepwise jumps [2], which can be detected by the search coil near the surface of the sample. Such sensitivity allows the MBN technique to be applicable in various nondestructive test (NDT) fields, such as residual stress [1,3,4], hardness [2,5], and anisotropy [6,7].

The root-mean-square (RMS) is a widely used feature of MBN, which is used for analysis in NDT measurements. Its amplitude is found to decrease with the increase in temperature [8,9,10]. For example, Wang et al. [8] and Guo et al. [9] experimentally showed a decreasing trend in the peak RMS amplitudes of MBN signals, which were measured for A3 and Q235 steels, respectively, as the increase in temperature was independent of the applied stress, and Altpeter [10] observed that the RMS amplitude of the compact cementite specimen disappeared at its Curie temperature. Since Barkhausen noise is originated from the magnetic properties of ferromagnetic material [11,12,13,14], and in turn, the magnetic properties are directly influenced by temperature, this leads to a direct influence of temperature on magnetic Barkhausen noise [15,16]. However, the temperature rarely independently affects the MBN signal. The environmental temperature may lead to a thermally induced stress where, for example, tens or even hundreds of MPa of stress values can be reached in a seamless track of high-speed railway [17,18]. Due to the sensitivity of MBN to stress [1,3,4,5], thermal stress could result in a noticeable RMS change [17]. Therefore, it is necessary to understand and distinguish the mechanism of the effects caused by temperature and thermal stress and evaluate these effects on MBN quantitatively.

The theoretical description of the Barkhausen effect is known to be a difficult task due to its random nature. To the best of our knowledge, few attempts have been made to quantitatively analyze the combined effects of temperature and thermal stress on the MBN measurement. However, progress has been made in recent years. The most notable attempt to mathematically describe the Barkhausen emission was made by Alessandro, Beatrice, Bertotti, and Montorsi (ABBM) [19], who proposed a model of the effect based on the stochastic process. The model was extended to the entire hysteresis loop by Jiles, Sipahi, and Williams (JSW) [11], who assumed the Barkhausen activity in a given time interval was proportional to the rate of change of magnetization. Subsequently, Jiles et al. [20] modified the differential susceptibility *dM*/*dH* as *dM_irr_*/*dH* to eliminate the influence of reversible magnetization that rarely induces Barkhausen activity. Lo et al. [12] used an extended hysteretic-stochastic model, introducing the magnetomechanical effect, to simulate the influence of stress on Barkhausen emission. Mierczak et al. [13] found the linear dependency of the reciprocal peak amplitude of MBN signal on stress and proposed a method for evaluating the effect of stress. Wang et al. [8] and Guo et al. [9] investigated the temperature effect of stress detection using MBN and proposed an analytical model based on the average volume of Barkhausen jump.

In this paper, the MBN model combined with the Jiles-Atherton (J-A) hysteresis model that has exerted latent capacity to introduce the effects of stress [21] and temperature [15,16] is adopted to study the theoretical correlations between Barkhausen emission and temperature. The methods to quantitatively evaluate the direct temperature effect only and the combined effect of temperature and thermal stress on MBN are presented. The rest of this paper organizes as follows. In Section 2, the temperature-dependent MBN models are proposed based on the J-A hysteresis model. In Section 3, the details about the verification experiments, including the specimen tempered procedure and the MBN sensor configuration, are explained. Both the performance and limitations of the proposed model are discussed in Section 4. Finally, the major findings of this study are discussed in Section 5.

## 2. The Effect of Temperature on Magnetic Barkhausen Noise

### 2.1. The Model of the Temperature Dependence of Hysteresis

According to the fundamental idea of the J-A model [22,23], the bulk magnetization *M* should be the sum of two parts, i.e., irreversible and reversible magnetization components:

(1) $$M=M_{rev}+M_{irr}$$

Irreversible and reversible magnetization components are given by

(2) $$M_{irr}=M_{an}-\delta k\frac{dM_{irr}}{dH_{e}}$$

(3) $$M_{rev}=c\left(M_{an}-M_{irr}\right)$$

where *M_an_* is the anhysteretic magnetization, and, e.g., in the case of isotropic materials, it is given by [24]

(4) $$M_{an}=M_{st}\left(\coth\left(\frac{H_{e}}{a}\right)-\frac{a}{H_{e}}\right)$$

where *H_e_* is the effective magnetic field intensity and is given by

(5) $$H_{e}=H+\alpha M$$

The saturation magnetization *M_st_*, the pinning factor *k*, the domain density *a*, domain coupling factor *α*, and the reversibility factor *c* are the key five parameters in the J-A model, and *δ* denotes the sign of d*H*/d*t*. In order to eliminate the unphysical negative susceptibility, the differential susceptibility relation given in Refs. [23,25] is employed here

(6) $$\frac{dM}{dH}=\frac{\chi_{M}}{k\delta -\alpha \chi_{M}}$$

where

(7) $$\chi_{M}=\delta_{m}\left(M_{an}-M\right)+k\delta c\frac{dM_{an}}{dH_{e}}$$

and

(8) $$\delta_{m}=\{\begin{array}{c} 0:\frac{dH}{dt}<0\;and\;M_{an}\left(H_{e}\right)-M\left(H\right)>0\\ 0:\;\frac{dH}{dt}>0\;and\;M_{an}\left(H_{e}\right)-M\left(H\right)<0\\ 1:otherwise\;\;\;\;\;\;\;\;\;\;\;\;\;\;\;\;\;\;\;\;\;\;\;\;\;\;\;\;\;\;\;\;\;\;\;\;\;\;\;\;\;\;\;\;\;\;\; \end{array}$$

The thermal effect can be incorporated into the J-A model Equations (1)–(8) by introducing thermal dependence of the five key microscopic hysteresis parameters. In this paper, the temperature-dependent J-A model has been extended based on our previous models [15,16] using a reference temperature instead of absolute zero and developing an equation for the temperature-dependent reversibility factor, *c*, which was previously assumed to be a constant. 

According to the Weiss theory of ferromagnetism, the spontaneous magnetization *M_s_* is the highest as the magnetic moments within a domain try to perfectly align when approaching absolute zero. As the temperature increases, it decreases until zero at the Curie point. Following an analogous argument to the spontaneous magnetization equation given in Refs. [15,16], the temperature dependence of saturation magnetization, *M_st_*, can be given as

(9) $$M_{st}\left(T\right)=M_{st}\left(T_{r}\right)\cdot {\left(\frac{T_{c}-T}{T_{c}-T_{r}}\right)}^{\beta_{1}}$$

where *M_st_*(*T_r_*) is the value of saturation magnetization at reference temperature (for example, 20 °C), which is more easily measured than that at 0 K, *T_c_* is the Curie temperature, and *β*_1_ is the material-dependent critical exponent according to mean-field theory. In case $T_{r}=0$ K, Equation (9) would turn to the original equation given in Refs. [15,16].

The domain wall pinning factor, *k*, is expected to vary with the exponential decay of coercive field with temperature in a ferromagnetic material according to the equation:

(10) $$k\left(T\right)=k\left(T_{r}\right)\cdot \exp\left[\frac{1}{\beta_{2}}\cdot \frac{T_{r}-T}{T_{c}}\right]$$

where *k*(*T_r_*) is the pinning factor at the reference temperature, and *β*_2_ is the critical exponent for the pinning constant.

The domain density, *a*, shows a similar exponential decay with temperature, which can be expressed as

(11) $$a\left(T\right)=a\left(T_{r}\right)\cdot \exp\left[\frac{1}{\beta_{3}}\cdot \frac{T_{r}-T}{T_{c}}\right]$$

where *a*(*T_r_*) is the domain density at the reference temperature, and *β*_3_ is the critical exponent for domain density and is generally approximated to be equal to *β*_2_.

The domain coupling, *α*, which represents the strength of magnetic interaction between domains in an isotropic material, can be expressed as

(12) $$\alpha =\frac{3a}{M_{st}}-\frac{1}{\chi_{an}^{\prime }}$$

At higher anhysteretic susceptibilities, $\chi_{an}^{\prime }$, the contribution of the second term to domain coupling is negligible, and, hence, substituting the expression for *M_st_* and *a* from Equations (9) and (11), respectively, yields as a first approximation

(13) $$\alpha \left(T\right)=\alpha \left(T_{r}\right)\cdot \exp\left[\frac{1}{\beta_{3}}\cdot \frac{T_{r}-T}{T_{c}}\right]\cdot {\left(\frac{T_{c}-T_{r}}{T_{c}-T}\right)}^{\beta_{1}}$$

where *α*(*T_r_*) is the domain coupling at the reference temperature.

The reversibility factor, c, is treated in an analogous way to that of domain coupling, *α*, and, for isotropic materials, is expressed as

(14) $$c=\frac{3a}{M_{s}}\chi_{in}^{\prime }$$

According to measurements [26], the initial susceptibility, $\chi_{in}^{\prime }$, also shows approximately exponential decay and can be expressed as a similar equation to Equation (11), and substituting the expression for *M_st_* and *a* from Equations (9) and (11), respectively, gives

(15) $$c\left(T\right)=c\left(T_{r}\right)\cdot \exp\left[\frac{1}{\beta_{3}\cdot \beta_{4}}\cdot \frac{T_{r}-T}{T_{c}}\right]\cdot {\left(\frac{T_{c}-T_{r}}{T_{c}-T}\right)}^{\beta_{1}}$$

where *c*(*T_r_*) is the reversibility factor at the reference temperature, and *β*_4_ is the additional critical exponent by considering the temperature-dependent initial susceptibility.

Therefore, the *M-H* hysteresis model of Equation (6) can be modified as

(16) $$\frac{dM\left(T\right)}{dH}=\frac{\chi_{M}\left(T\right)}{k\left(T\right)\delta -\alpha \left(T\right)\chi_{M}\left(T\right)}$$

where

(17) $$\chi_{M}\left(T\right)=\delta_{m}\left[M_{an}\left(T\right)-M\left(T\right)\right]+k\left(T\right)\delta c\left(T\right)\frac{dM_{an}\left(T\right)}{dH_{e}\left(T\right)}$$

### 2.2. The Magnetomechanical Hysteresis Model

When a ferromagnetic material is subjected to the action of elastic stress (*σ*) in an applied magnetic field (*H*), the magnetization (*M*) of the material is dominated by an effective field, *H_e_*, which can be expressed as [12,21,27]

(18) $$H_{e}=H+\alpha M+H_{\sigma }$$

where *H_σ_* represents the equivalent magnetic field induced by the stress. This equivalent field results from the magnetoelastic coupling and is given by

(19) $$H_{\sigma }=\frac{3}{2}\frac{\sigma }{\mu_{0}}\left(\cos^{2}\theta -\nu sin^{2}\theta \right)\left(\frac{\partial \lambda }{\partial M}\right)$$

where *ν* is the Poisson’s ratio, *θ* is the angle between the stress axis and the direction of *H_σ_*, and *λ* is the bulk magnetostriction, whose partial differential with respect to magnetization is determined by fitting *λ* ≈ *a* + *bM*^2^ [13,28] from the experiment. When the direction of stress is parallel to that of magnetization, Equation (19) can be rewritten as

(20) $$H_{\sigma }=\frac{3\sigma }{\mu_{0}}bM$$

Hence, Equation (18) can be simplified as

(21) $$H_{e}=H+\tilde{\alpha }M$$

where

(22) $$\tilde{\alpha }=\alpha +\frac{3\sigma b}{\mu_{0}}$$

Therefore, taking the effect of stress into account, Equation (6) will be improved as

(23) $$\frac{dM\left(\sigma \right)}{dH}=\frac{\chi_{M}}{k\delta -\tilde{\alpha }\chi_{M}}$$

### 2.3. The Effect of Temperature on Magnetic Barkhausen Noise

The Barkhausen emissions caused by the discontinuous magnetization changes inside ferromagnetic material with stochastic nature have been modeled based on the J-A model previously [11,12,13,14,20]. According to the basic model, the sum of Barkhausen jumps in the given period Δ*t* is proportional to the total variation of irreversible magnetization following the equation:

(24) $$M_{JS}=\gamma \cdot \frac{dM_{irr}}{dt}\cdot \Delta t=\gamma \cdot \frac{dM_{irr}}{dH}\cdot \frac{dH}{dt}\cdot \Delta t$$

where *γ* is a coefficient with respect to the irreversible magnetization, and it can be further subdivided into the number of Barkhausen jumps events *N* and the average size of discontinuous jumps $\langle M_{disc}\rangle$.

(25) $$\gamma =\frac{d\left(N\langle M_{disc}\rangle \right)}{dM_{irr}}$$

The average size of discontinuous jumps, $\langle M_{disc}\rangle$, is likely weakly related to the irreversible magnetization. The number of Barkhausen events *N* is considered as a stochastically fluctuating function, and the behavior of Barkhausen events is assumed to follow a Poisson distribution [11,20]

(26) $$N_{t}=N_{t-1}+\delta_{rand}\sqrt{N_{t-1}}$$

where *δ_rand_* is a random number lying in the range ±1.47.

The differential expression of Barkhausen jumps is given as [14,20]

(27) $$\frac{dM_{JS}}{dt}=\frac{dM_{irr}}{dH}\cdot \frac{dH}{dt}\cdot \langle M_{disc}\rangle \cdot \frac{dN}{dM_{irr}}$$

#### 2.3.1. Case 1: The Direct Effect of Temperature Only

When the temperature effect is taken into account, the thermal energy influences the magnetization behavior. It leads to changes in magnetic properties, such as susceptibility, coercivity, and hysteresis loss. It further affects the Barkhausen jumps as

(28) $$\frac{dM_{JS}\left(T\right)}{dt}=\frac{dM_{irr}\left(T\right)}{dH}\cdot \frac{dH}{dt}\cdot \langle M_{disc}\rangle \cdot \frac{dN}{dM_{irr}\left(T\right)}$$

where

(29) $$\frac{dM_{irr}\left(T\right)}{dH}=\frac{M_{an}\left(T\right)-M_{irr}\left(T\right)}{k\left(T\right)\delta }\left(1+\frac{\alpha \left(T\right)dM\left(T\right)}{dH}\right)$$

In Equation (28), if the rate of change of applied magnetic field *dH*/*dt* with time is consistent during the measurements under various temperatures, the Barkhausen jumps are dominated by the differential susceptibility of irreversible magnetization *dM_irr_*(*T*)/*dH* as the rest part on the right-hand represents the random behavior of the model. It is known that the maximum value of Barkhausen noise occurs at coercivity point *H_c_* [12,13] so that the peak amplitude of MBN can be written as

(30) $$MBN_{p}=\chi_{H_{c}}^{\prime }\left(\frac{dH}{dt}{|}_{H_{c}}\right)\cdot \gamma \cdot \Delta t$$

where $\chi_{H_{c}}^{\prime }$ is the differential susceptibility of irreversible magnetization at the coercivity point. It is known that in a soft ferromagnetic material, the maximum differential susceptibility of irreversible magnetization $\chi_{H_{c}}^{\prime }$ can be approximated by hysteresis differential susceptibility $\chi_{an}^{\prime }$ [12,13]. *γ* represents the random behavior of the model. As the predicted and measured RMS of the MBN are compared in this study, the stochastic fluctuation caused by Poisson distribution is replaced by the expectation after averaging. Using Equation (12), we arrive at

(31) $$\frac{1}{\chi_{H_{c}}^{\prime }\left(T\right)}-\frac{1}{\chi_{H_{c}}^{\prime }\left(T_{r}\right)}=\frac{3a\left(T_{r}\right)\xi \left(T\right)}{M_{st}\left(T_{r}\right){\left(\frac{T_{c}-T}{T_{c}-T_{r}}\right)}^{\beta_{1}}}-\alpha \left(T_{r}\right)\xi \left(T\right)$$

where

(32) $$\xi \left(T\right)=\exp\left[\frac{1}{\beta_{3}}\cdot \frac{T_{r}-T}{T_{c}}\right]\cdot {\left(\frac{T_{c}-T}{T_{c}-T_{r}}\right)}^{\beta_{1}}-1$$

When the rate of change of applied field with time is determined, and the random behavior is ignored, the temperature-dependent peak values of MBN, *MBN_p_*(*T*), deduced from Equations (30) and (31), can be given as:

(33) $$\frac{1}{MBN_{p}\left(T\right)}-\frac{1}{MBN_{p}\left(T_{r}\right)}=\kappa \left[\frac{3a\left(T_{r}\right)\xi \left(T\right)}{M_{st}\left(T_{r}\right){\left(\frac{T_{c}-T}{T_{c}-T_{r}}\right)}^{\beta_{1}}}-\alpha \left(T_{r}\right)\xi \left(T\right)\right]$$

where *κ* is a constant coefficient about the rate of applied field change and the average irreversible magnetization coefficient at the coercivity point. In the case that the environmental temperatures are far from the Curie Temperature, we expand the binomial series and exponential function using the Tylor series. Omitting the high-order and infinitesimal items, Equation (33) can be rewritten as

(34) $$\frac{1}{MBN_{p}\left(T\right)}-\frac{1}{MBN_{p}\left(T_{r}\right)}=\kappa \left[A+B\times T\right]$$

where *A* and *B* are constants since all the parameters are determined and given as

(35) $$A=\frac{3a\left(T_{r}\right)}{M_{st}\left(T_{r}\right)\cdot \beta_{3}}-\frac{\alpha \left(T_{r}\right)}{\beta_{3}}$$

(36) $$B=\frac{\alpha \left(T_{r}\right)}{T_{c}\cdot \beta_{3}}-\frac{3a\left(T_{r}\right)}{M_{st}\left(T_{r}\right)\cdot T_{c}\cdot \beta_{3}}$$

Equation (34) shows the linear tendency of the reciprocal MBN peak value, representing the effect of temperature on Barkhausen noise.

#### 2.3.2. Case 2: The Combined Effects of Temperature and Thermal Stress

Such an effect of temperature on magnetic properties is a direct one, but generally, an indirect effect exists. Namely, modification of temperature may induce stresses in solid structures that would change magnetic properties as well. These thermal-induced stresses can be classified into two types: type 1 is caused by different parts of a long or large structure exposed to different environmental temperatures, such as railway

(37) $$\varepsilon_{T1}=\zeta_{T}\cdot \left(T_{1}-T_{2}\right)$$

and type 2 is resulted from two materials with different coefficients of thermal expansion (CTE) fixed together, such as multilayer plate

(38) $$\varepsilon_{T2}=\left(\zeta_{T1}-\zeta_{T2}\right)\cdot \left(T_{ref}-T\right)$$

where *ε_T1_* and *ε_T2_* are the thermal strains induced by the types 1 and 2, respectively, and *ζ_T1_* and *ζ_T2_* are the larger and the smaller coefficients of thermal expansion of two materials, respectively, and *T_ref_* is the reference temperature [29]. 

The thermal stress, σ, could be inferred from the thermal strain below elastic limitation [30]

(39) $$\left[\begin{array}{c} \sigma_{xx}\\ \sigma_{yy}\\ \begin{array}{c} \sigma_{zz}\\ \sigma_{xy}\\ \begin{array}{c} \sigma_{xz}\\ \sigma_{yz} \end{array} \end{array} \end{array}\right]=\frac{E}{\left(1+\nu \right)\left(1-2\nu \right)}\left[\begin{array}{cccccc} 1-\nu & \nu & \nu & 0 & 0 & 0\\ \nu & 1-\nu & \nu & 0 & 0 & 0\\ \nu & \nu & 1-\nu & 0 & 0 & 0\\ 0 & 0 & 0 & \left(1-2\nu \right)/2 & 0 & 0\\ 0 & 0 & 0 & 0 & \left(1-2\nu \right)/2 & 0\\ 0 & 0 & 0 & 0 & 0 & \left(1-2\nu \right)/2 \end{array}\right]\left[\begin{array}{c} \varepsilon_{xx}\\ \varepsilon_{yy}\\ \begin{array}{c} \varepsilon_{zz}\\ 2\varepsilon_{xy}\\ \begin{array}{c} 2\varepsilon_{xz}\\ 2\varepsilon_{yz} \end{array} \end{array} \end{array}\right]$$

where *σ_xx_* and *ε_xx_* are the *x*-axis component of thermal stress and strain, *E* is Young’s modulus, and *ν* is Poisson’s ratio. Assuming there is no fixed constraint along the z-axis, for an isotropic lamination specimen, *σ_zz_*, *σ_xz_*, *σ_yz_* are approximately equal zero, *σ_xy_* = *τ_xy_*, and *ε_xy_* = *γ_xx_*/2; hence, Equation (36) can be simplified as

(40) $$\left[\begin{array}{c} \sigma_{xx}\\ \sigma_{yy}\\ \tau_{xy} \end{array}\right]=\frac{E}{\left(1+\nu \right)\left(1-2\nu \right)}\left[\begin{array}{c} 1-\nu \\ \nu \\ 0 \end{array}\begin{array}{c} \nu \\ 1-\nu \\ 0 \end{array}\begin{array}{c} 0\\ 0\\ \left(1-2\nu \right)/2 \end{array}\right]\left[\begin{array}{c} \varepsilon_{xx}\\ \varepsilon_{yy}\\ \gamma_{xy} \end{array}\right]$$

Assuming the direction of magnetization is parallel to the *y*-axis, the stress along the *y*-axis calculated by Equation (40) will be substituted into Equation (20) for further magnetic simulation using Equation (23). Considering the combined effect of temperature and thermal-induced stress, Equation (23) is rewritten as

(41) $$\frac{dM\left(T\right)}{dH}=\frac{\chi_{M}\left(T\right)}{k\left(T\right)\delta -\tilde{\alpha }\left(T\right)\chi_{M}\left(T\right)}$$

Substituting the new equation of differential susceptibility of magnetization into Equation (29) would obtain Barkhausen noise expression influenced by the joint actions of temperature and thermal-induced stress similar to Equation (28). It would represent the Barkhausen jump behavior at a given temperature. However, we are more concerned with the extent to which the temperature and thermal-induced stress impact Barkhausen noise. Following an analogous argument to the reciprocal MBN peak value influenced by temperature exclusively, the reciprocal MBN peak value impacted by the combined effects of temperature and thermal stress is given by the following expression

(42) $$\frac{1}{MBN_{p}\left(T\right)}-\frac{1}{MBN_{p}\left(T_{r}\right)}=\kappa_{1}\left[A+B\times T-\frac{3b\sigma \left(T\right)}{\mu_{0}}\right]$$

where *κ*_1_ is a constant coefficient analogous to *κ*. 

The improved MBN model, including the direct effect of temperature and the indirect effect of thermal stress, provides a way to investigate the effects of temperature on the MBN signals. The application scope of the proposed model is not limited to the case in this study. It is also adequate for modeling MBN with the multiphysics problems involving temperature and stress if the magnetic properties and the magnetostrictions can be determined.

## 3. Experiments

### 3.1. The MBN Experiments Considering the Direct Effect Only

The MBN experiments that study the direct effect of temperature itself on MBN are conducted on the lamination disc of non-oriented (NO) grain silicon steel with 0.50 mm in thickness and 30 mm in diameter. Such specimen sizes could facilitate fast and evenly heating/cooling of the whole body of the sample. Compared with grain-oriented (GO) silicon steel, the NO specimen can be considered an isotropic material in magnetic and mechanic properties.

**Figure 1:**
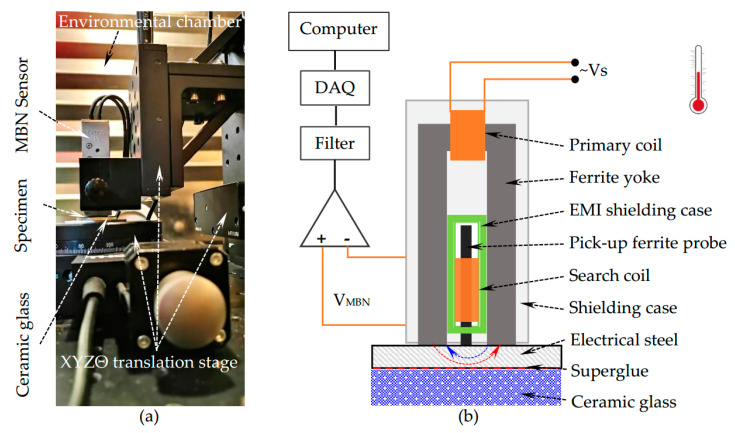
(**a**) The magnetic Barkhausen noise (MBN) experimental set-up; (**b**) The schematic diagram of the Barkhausen sensor.

In this study, the Barkhausen noise measurements are carried out in the environmental chamber HC4033 from Vötsch. It uses the compressor to refrigerate and the fan to ventilate, which might introduce undesired vibration and electromagnetic interference. Hence, the S1-16-12-01 type MBN sensor supplied by Stresstech with shielding case and good stability could reduce electromagnetic interference. Besides, the sensor is assembled on a motorized XYZΘ translation stage from Thorlabs to move the sensor to the center of the specimen in precise control and steadily contact the sample surface. The measurement set-up is mounted on a non-magnetic breadboard, placed on a shock mitigation frame to further reduce vibration interference. The experimental set-up is cooled and heated together with the sample. There are two test holes in the chamber used to connect the experimental set-up in the chamber to the control and data acquisition (DAQ) systems out of the chamber. The sensor is communicated with the computer through the Microscan 600 system, which could control the start, stop, magnetizing frequency, etc., and acquire the MBN data. The experimental set-up and the schematic diagram of the Barkhausen sensor are presented in Figure 1. During measurement, the sinusoidal current is fed into the primary coil to generate magnetic flux in the ferrite yoke, which forms magnetic flux closure with the test sample. The Barkhausen emissions from the magnetized section of the tested sample are detected in the form of voltage pulses induced in the searching coil winding on a ferrite probe. The magnetizing frequency and voltage used in the measurements are set to 50 Hz and 10 V, respectively. The pick-up coil’s output voltage is subsequently amplified with the low noise AD797 operational amplifier and digitized by the Microscan 600 system with a sampling frequency of 2.5 MHz.

In these MBN experiments, the sample and the measurement set-up are refrigerated from 20 °C to −40 °C with 10 °C temperature interval and then heated up to 60 °C with 10 °C increments. The temperatures are set step by step. At each set temperature point, such as 20 °C, 10 °C, and 0 °C, the measurement will not be implemented until the temperature is steady for more than 10 min to evenly cool or heat the sample and avoid the effect of temperature variation [31]. At each set temperature point, eight cycles of Barkhausen noise signal are measured, and the mean value of RMS is obtained. The entire process is repeated five times to reduce the measurement error.

Before these MBN experiments, the specimens are annealed at 400 °C for two hours to relieve the residual stress. The quasi-static hysteresis of a sheet specimen of the same material at different temperatures is measured to determine the key parameters of the temperature-dependent J-A model. The key parameter values are determined by the hybrid GA-PSO algorithm (GA and PSO represent Genetic Algorithm and Particle Swarm Optimization, respectively), and the results are listed in Table 1.

### 3.2. The MBN Experiments Considering the Combined Effects

**Figure 2:**
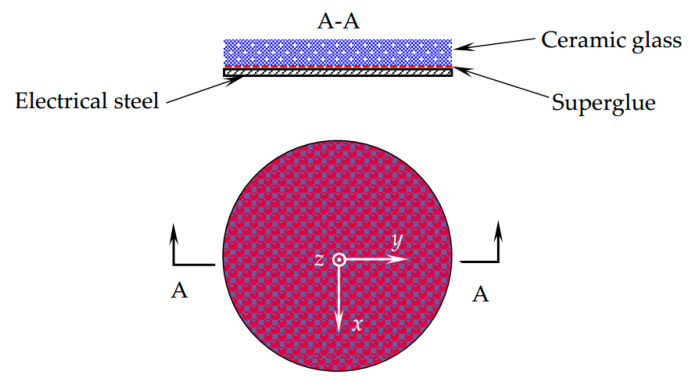
The thermally induced stress structure.

In the MBN experiments that study the combined direct and indirect effects on MBN, the NO silicon steel disc with 0.50 mm thickness is glued to a ceramic glass disc (Schott Zerodur), whose CTE ($1\;\times \;10^{-7}\;$°C^−1^) is much smaller than NO steel ($11.9\;\times \;10^{-6}$ °C^−1^), at room temperature (20 °C). The experimental conditions related to this work can be described via the type 2 thermal stresses, where two components with different CTEs are fixed together at the reference temperature. The multilayer structure shown in Figure 2 could induce thermal stress when the temperature changes due to the considerable difference in CTE between the two materials. 

Similar to the experiments described in the previous subsection, the new sample is cooled from 20 °C to −40 °C and heated up to 60 °C with 10 °C intervals. The magnetizing frequency and voltage used in the measurements are set to 50 Hz and 5 V, respectively. The measurement process is repeated five times as well. Prior to these MBN experiments, the key parameter of magnetostriction (*λ*) is measured. Its value, together with the values of Young’s Modulus (*E*) and Poisson’s Ratio (*ν*), are listed in Table 1. 

## 4. Results and Discussion

**Figure 3:**
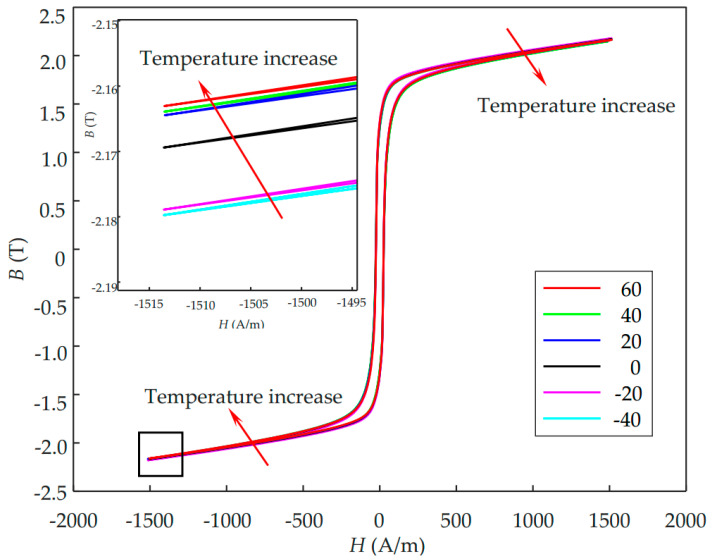
Hysteresis loops of 0.50 mm thickness non-oriented (NO) electrical steel at various temperatures. The inset shows an enlarged view of the negative tips of hysteresis loops.

The magnetic hysteresis loops of the NO silicon steel sheet are measured using a computer-controlled hysteresis loop tracer at a quasi-DC field of 5 MHz. The measurement system is subject to various temperatures that are controlled by the environmental chamber. The experimental results of hysteresis loops of 0.5 mm NO electrical steel at different temperatures are illustrated in Figure 3. It can be found in the inset figure that the maximum absolute values of induced magnetic density (B) decrease with an increase in the temperature. The hybrid GA-PSO algorithm is used to identify the temperature-dependent J-A parameters by fitting the hysteresis loops in Figure 3, and the fitted parameters are listed in Table 1.

### 4.1. The MBN Experiments Considering the Direct Effect Only

**Figure 4:**
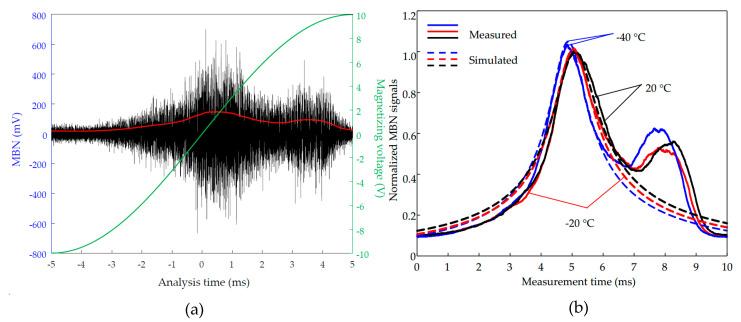
(**a**) Illustration of raw Barkhausen bursts and the corresponding root-mean-square (RMS) envelope. (**b**) The simulated and measured MBN signal envelops for 0.5 mm thickness NO electrical steel under various temperatures.

The typical raw MBN signal measured for the NO steel is plotted in Figure 4a. The RMS feature of the MBN signal is extracted for analysis. The experimental MBN signals along the y-axis at −40 °C, −20 °C, and 20 °C as examples are shown in Figure 4b, and the corresponding simulated MBN signals using Equations (24) and (28) are plotted at the related locations of experimental ones. All the simulated and measured MBN signals are normalized by the maximum amplitudes of the simulated and measured MBN signals at 20 °C, respectively. It can be found that the highest amplitude of the simulated MBN signals is consistent with the measured ones. It indicates that the proposed temperature-dependent MBN model is adequate to predict the RMS profile of MBN under various temperatures accurately. The RMSs of the measured MBN signals show one more peak than those of the simulation. It is caused by the mixed texture of grain in the NO steel. Compared with the grain-oriented (GO) electrical steel, which has the unique Goss texture ({110}<001>), resulting in the alignment of the easy axis (<001>) to the rolling direction, the non-oriented (NO) electrical steel consists of mixed texture. It is usually considered isotropic on the macroscopic scale. But the NO steels are usually manufactured under two-stage cold rolling with intermediate annealing. After the first cold rolling, the annealing could recrystallize and decarburize the steel. After the second cold rolling, the annealing can remove residual stress and obtain the desired random orientation of grain growth [35]. During the process, there are mixed textures, including textures along the easy axis, such as <100>, and textures along the hard axis, such as <111>. The domain, including the former textures, is magnetically softer than the domain containing the later one, which inherits to result in two peaks in MBN signal, but much less pronounced than in the GO steel. Hence, in the simulation, we consider the NO electrical steel as an isotropic material, and its magnetic properties are modeled according to the measured hysteresis loop. Besides, the maximum peak values of measured MBN signals appear around the coercivity point corresponding to the prediction (see Figure 4). Therefore, the comparison of the maximum peak of simulated and measured results could be used to verify the feasibility of the model.

**Figure 5:**
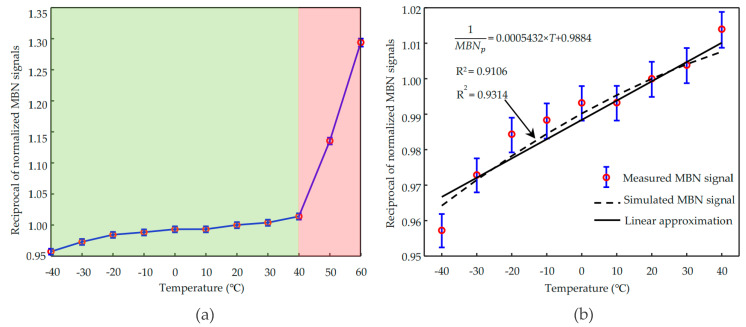
(**a**) The reciprocal MBN peak amplitude as a function of temperature measured from 0.5 mm thickness NO electrical steel. (**b**) Dependence of reciprocal MBN peak value on temperature approximated with a linear function.

To evaluate the relationship between the MBN signal and the temperature quantitatively, the reciprocals of maximum peak RMS values of the measured MBN are normalized by that at 20 °C and plotted in Figure 5a. It can be found that the values at 50 °C and 60 °C show an unusually steep rise. When the environmental temperature increases over 50 °C, even 60 °C, the temperature inside the sensor could be higher than the operating temperature. The primary coil operation will heat the sensor and can lead to an internal temperature higher than 80 °C. Generally, the Curie temperature of ferrite is around 100 °C, and its magnetic properties will sharply degrade when it is approaching its Curie point. Besides, the maximum operating temperature of the operational amplifier inside the sensor is 80 °C. Therefore, the measured MBN signals at 50 °C and 60 °C are eliminated in comparison with the simulated results. 

The predicted relation between MBN signal and temperature using Equation (33) is plotted in Figure 5b together with the measured results. It can be found that the dependence of the reciprocal peak amplitude of the MBN signal on the temperature obtained from experiments corresponds with the simulated one with a coefficient of determination higher than 0.93. For a ferromagnetic material with a much higher Curie temperature than the environmental temperature, such as iron (770 °C), the simplified Equation (34) for Equation (33) indicates that the dependence of reciprocal MBN peak amplitude on temperature is approximated with a linear function, as shown in Figure 5b. The linear approximation of Equation (34) in the normal environmental temperature range is consistent with the experimental results (*R*^2^ higher than 0.91).

### 4.2. The MBN Experiments Considering the Combined Effect

One of the factors that limit the applicability of the J-A magnetomechanical model using Equations (41), (29), and (28) to simulate the MBN signal is the domain coupling factor *α*. Its value is so small that it can easily become lower than zero with stress using Equation (22). Therefore, there is only a limited temperature range that allows the applicability of this multiphysics MBN model. It is necessary to mention that the model will work better in a magnetically harder material. In fact, to quantitatively evaluate the effect of temperature on the MBN signal, our main concern is the extent to which the temperature and corresponding thermal stress impact Barkhausen noise. Even for those harder magnetic materials that could calculate the MBN envelopes, their peak amplitudes will be further represented as a temperature function given in Equation (42). 

In the previous subsection, the reciprocal MBN peak amplitudes influenced by the direct effect of temperature are approximated as a linear function of temperature. Hence, Equation (34) can be rewritten as

(43) $$\frac{1}{MBN_{p}\left(T\right)}=p_{1\;}\times T+c$$

where *p*_1_ and *c* are constant coefficients. The last item of Equation (42) is also proportional to temperature if the coefficient *b* is constant. Hence, the characteristic of reciprocal MBN peak value is the linear superposition of two linear equations

(44) $$\frac{1}{MBN_{p}\left(T\right)}=p_{T\;}\times T+p_{\sigma \;}\times T+c$$

where *p_T_* and *p_σ_* are the constant coefficients for the direct and indirect effect of temperature, respectively.

**Figure 6:**
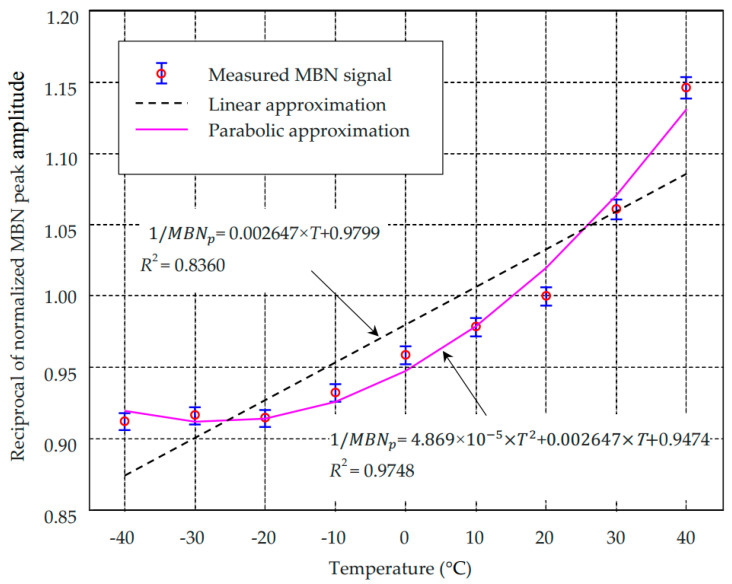
The approximation of reciprocal MBN peak amplitude as a parabolic function of temperature.

**Figure A1:**
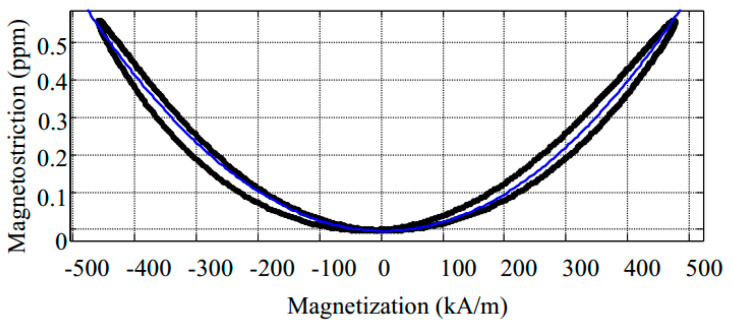
The magnetostriction as a function of magnetization for the 0.5 mm thickness NO electrical steel.

The approximated results of the reciprocal MBN signal as a linear function of temperature using Equation (41) are plotted in Figure 6 together with the measured results. It can be seen that the reciprocal peak value of the measured MBN signal exhibits a clear rising trend for increasing temperature, which is consistent with the prediction of Equation (42) due to the positive value of magnetostriction coefficient *b* (2.56 × 10^−18^ m^2^/A^2^ determined by the parabolic fitting measured λ-M curve as plotted in Figure A1). The fitting coefficient (0.002647) is much larger than that in Figure 5b (0.0005432), even with the lower excitation voltage. It indicates that the combined effect of temperature and thermal stress on the MBN signal is much more significant than the direct effect of temperature only. It can also be found that the fitting goodness of *R*^2^ (0.8360) is lower than that in Figure 5b. The reason for that being the magnetostriction coefficient *b* is rarely a constant.

In general, the magnetostriction curves, for example, reported for carbon steels [36] and electrical steels [37,38], have shown that the parabolic approximations of *λ*-*M* curves change with stresses, resulting in the different values of magnetostriction coefficient *b*. Considering the empirical equation of magnetostriction as a function of magnetization [21,27]

(45) $$\lambda \approx b_{0}+\left(b_{1}+b_{2}\sigma \right)M^{2}$$

where *b_0_*, *b*_1_, and *b*_2_ are magnetostriction coefficients. Equations (39) and (41) can be rewritten as

(46) $$\frac{1}{MBN_{p}\left(T\right)}-\frac{1}{MBN_{p}\left(T_{r}\right)}=\kappa_{1}\left[A+B\times T-\frac{3b_{2}\sigma^{2}\left(T\right)+3b_{1}\sigma \left(T\right)}{\mu_{0}}\right]$$

(47) $$\frac{1}{MBN_{p}\left(T\right)}=p_{1\;}\times T^{2}+p_{2\;}\times T+c$$

The measured reciprocal of MBN peak amplitude is parabolically approximated using Equation (47), as plotted in Figure 6. It can be found that the dependence of the reciprocal of MBN peak amplitude on the temperature obtained from experiments corresponds with the simulated one with a coefficient of determination higher than 0.97. It implies that the proposed parabolic dependency of 1/*MBN_p_* on temperature can be applied to evaluate the combined effect of temperature and thermal stress on MBN quantitatively.

In the normal environmental temperature, the thermal stress is usually in the elastic stress range of material. The dependence of MBN peak amplitude on temperature can also be approximated by parabolic function within this range. Therefore, Equations (46) and (47) complicate the relation between MBN and temperature rather than simplifying it. Besides, it is difficult to distinguish the direct and indirect effects of temperature due to the complicated relation and the difficulty in identifying those magnetostriction coefficients. To simplify the evaluation function, we adopt the method proposed in Ref. [13] to linearly approximate the dependence of reciprocal MBN peak amplitude on temperature. If the high-order term is eliminated, the coefficient of the equation is the same as the linear approximation, which has been proven to have relatively low fitting goodness. Therefore, the parabolic fitting magnetostriction coefficient *b* has various values rather than a constant.

It has been experimentally shown [36,37,38] that within the elastic limit, the maximum value of magnetostriction *λ* at a given low magnetization *M* presents approximately a linear increase with the increasing compressive stress and the decreasing tensile stress, respectively. But the linear approximations show a larger slope under compression than that under tension [37,38]. Somkun [38] has measured the peak-to-peak values of magnetostriction for 0.5 mm thick NO steel cut along the rolling direction under sinusoidal magnetization at 1.00 T. If we employ a piecewise linear function to fit the measured results under compression and tension respectively, the slope fitting under compression (−0.3336) is about 4.05 times under tension (−0.08235), which can be used to approximately represent the ratio of magnetostriction coefficient *b* under compression and tension. Hence, we consider evaluating the effect of the temperature higher than the reference temperature, for which corresponding thermal stress is compressive, and the temperature lower than the reference temperature, for which the corresponding thermal stress is tensile, separately. If we define the temperature higher than the reference temperature as high temperature and that lower than reference as low temperature, the Equation (47) can be further rewritten as piecewise linear functions and calibrated with the value at reference temperature (20 °C in this study) as there is no stress involving in the measurement at the reference temperature, and the measured MBN amplitude at the reference temperature is the benchmark value of normalization. The normalized reciprocal MBN peak value passing through the reference point is given as

(48) $$\{\begin{array}{c} \frac{1}{MBN_{pH}\left(T\right)}=(p_{H\sigma }+p_{T})\times \left(T-T_{ref}\right)+1\;\;\;\;\;\left(T\geq T_{ref}\right)\\ \frac{1}{MBN_{pC}\left(T\right)}=(p_{C\sigma }+p_{T})\times \left(T-T_{ref}\right)+1\;\;\;\;\;(T<T_{ref}) \end{array}$$

where *p_Hσ_* and *p_Cσ_* are the slopes related to the thermal stresses caused by high temperature and low temperature, respectively, and *p_T_* is the temperature coefficient similar to Equation (44).

**Figure 7:**
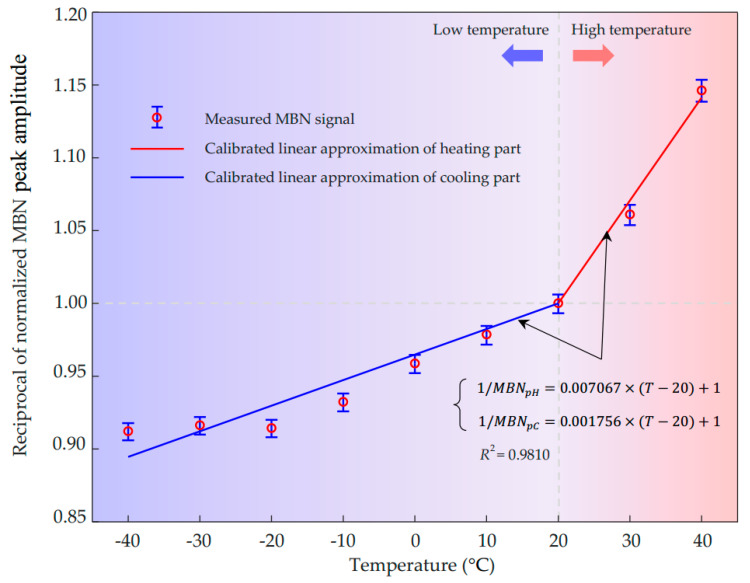
Dependence of reciprocal MBN peak value on temperature approximated with linear functions.

The reciprocal MBN peak amplitude as linear functions of temperature using Equation (48) fitting to the measured results (with a fitting goodness *R*^2^ higher than 0.98) is plotted in Figure 7. It implies that the simplified practice model can be applied to evaluate the combined effect of temperature on MBN peak amplitude. The ratio of the slopes under high and low temperatures shown in Figure 7 is 4.02, which is close to the ratio of the fitting slopes for magnetostriction under compressive and tensile stresses (4.05). The difference may owe to the direct effect of temperature in addition to the effect of thermal stress and the errors caused by fitting and measurement.

For a new ferromagnetic material influenced by temperature, if the prior knowledge of the temperature-dependent hysteresis and stress-dependent magnetostriction has been obtained, we could simulate the MBN profile influenced by temperature through Equation (28). In practice, to obtain linear functions of temperature, we could measure two or more data points and deduce the linear function of temperature by using Equations (34), (42), (43), and (48). The MBN peak amplitude at reference temperature (e.g., 20 °C) needs to be measured to determine the benchmark value at first. At least another point is needed to obtain for linear approximation the reciprocal MBN peak value vs. temperature. Suppose there is only the effect of temperature itself involving in the experiments; in this case, the linear fitting function could characterize the dependence of reciprocal MBN peak value on temperature and quantitatively evaluate the effect of temperature on the MBN signal. For example, as shown in Figure 5b, temperature heating from −40 °C to 40 °C results in an increase of 4.49% in the reciprocal of MBN peak value, which means the MBN peak amplitude decreases 4.60% in this temperature range.

In the case of thermal stress involvement, one or more points apart from reference one should be measured either in a high-temperature range or a low-temperature range. Taking the points in the high-temperature range shown in Figure 7, for instance, a linear function passing through the reference point (20 °C) could be obtained by Equation (48). This linear function could represent the dependency of reciprocal MBN peak amplitude on heating temperature. There are two methods to determine the relationship between 1/*MBN_p_* and temperature in the low-temperature range. The simplest one is to measure one or more points in the low-temperature range and use a linear function to fit them as the blue line plotted in Figure 7. For another method, calculating the coefficients of thermal stress *p_Hσ_* and temperature *p_T_* is required using Equations (42) and (44). The computed coefficients caused by thermal stress and temperature are about 7.057 × 10^−3^ and 1.000 × 10^−5^, respectively. Since the ratio of the magnetostriction coefficient *b* under compression and tension is around 4.05, we can obtain the coefficient caused by thermal stress in the low-temperature range, *p_Cσ_*, with a value of 0.001742. Consequently, the linear function slope for evaluating the dependence of 1/*MBN_p_* on temperature is 1.752 × 10^−3^, which is closely approaching the slope of the best linear fitting function (1.756 × 10^−3^). It indicates that this method is feasible to evaluate the effect of temperature on the MBN peak amplitude quantitatively. The piecewise linear dependency of the reciprocal MBN peak amplitude on temperature is concluded.

The environmental temperature heating from −40 °C to 40 °C results in an increase of 27.54% in the reciprocal of MBN peak value. Therefore, its effect should be considered in precise evaluation using the MBN method, such as evaluating residual stress and case depth. To analyze the low and high-temperature range separately, the environmental temperature cooling from reference temperature 20 °C to −40 °C leads to a decrease of 10.54% in 1/*MBN_p_*, where we could infer the MBN peak amplitude increases by 11.78%. While the temperature heating from 20 °C to 40 °C causes a sharper increase of 14.13% in 1/*MBN_p_*, which means the peak amplitude of the MBN signal attenuates by 12.38% quickly.

## 5. Conclusions

This paper has considered both the direct effect of temperature and the indirect effect of thermal stress. If only the direct effect is involved, the extended MBN model based on temperature-dependent hysteresis is proposed. The relationship between the reciprocal MBN peak amplitude and temperature, which has been further simplified as the linear function to evaluate the dependence of MBN peak amplitude on temperature quantitatively, is deduced from the temperature-dependent MBN model. While considering the combined effects of temperature and thermal stress, the multiphysics MBN model has been presented, and, based on this model, the parabolic dependence of the reciprocal MBN peak value on temperature is given. Practical piecewise linear functions are then presented to approximate the dependence according to the finding that the magnetostriction coefficients under compression and tension are different.

Temperature experiments for magnetic hysteresis measurements are conducted before MBN experiments. The temperature-dependent parameters of the J-A model are determined by using the hybrid GA-PSO algorithm. When the direct effect of temperature itself is exclusively involved, the measured peak value of MBN signals fits with the simulated MBN envelopes well, and the reciprocal of the peak amplitude of the MBN signal has experimentally shown the linear variation with temperature corresponding with the predicted results. The linear dependency would be useful for the quantitative evaluation of temperature on the MBN signal. In this case, temperature heating from −40 °C to 40 °C results in an increase of 4.49% in the reciprocal MBN peak value. 

While in addition to the direct effect, the indirect effect of thermal stress is involved. The measured reciprocal of the peak amplitude of Barkhausen emission has presented parabolic dependency on temperature, which is consistent with the predicted tendency. The parabolic relation is further simplified by a piecewise linear function at temperatures higher and lower than the reference temperature. It has been proven to be feasible to evaluate the combined effect quantitatively. The environmental temperature cooling from the reference temperature 20 °C to −40 °C leads to a decrease of 10.54% in 1/*MBN_p_*. Whereas the temperature heating from 20 °C to 40 °C causes a sharper increase of 14.13% in 1/*MBN_p_*. The methods to obtain the piecewise linear function used to evaluate the joint effects of temperature and thermal stress have been proposed. To achieve the evaluation of material and mechanical properties using the MBN method with high accuracy, the effect of temperature on the MBN signal should be considered in the calibration process of MBN measurement. Moreover, the MBN is a potential method in structural health monitoring. But the temperature compensation for the monitoring data under various temperatures is a subject remaining to be researched, and the proposed practical method would be possible to solve this problem. 

## Figures and Tables

**Table 1:** The key parameters of the J-A hysteresis model for 0.5 mm NO steel.

J-A Parameters (at 20 °C)	Values	Sources	Mechanical Parameters	Values	Sources
Saturation magnetization, *M_st_*	$1.7157\;\times 10^{6}\;$(A/m)	Measured and identified by hybrid GA-PSO algorithm	CTE of electrical steel, *ζ_T1_*	$11.9\;\times 10^{-6}$ (°C^−1^)	Ref. [32]
Pining parameter, *k*	103.8603 (A/m)		CTE of Ceramic glass, *ζ_T2_*	$1.0\;\times \;10{\;}^{-7}$(°C^−1^)	Ref. [33]
Domain density, *a*	65.5559 (A/m)		Young’s Modulus of NO steel, *E*	205 (GPa)	Ref. [34]
Coupling factor, *α*	$1.2492\times 10^{-4}$		Poisson’s Ratio of NO steel, *ν*	0.28	
Reversibility parameter, *c*	0.6799		Magnetostriction coefficient, *b*	2.56 × $10^{-18}$ (m^2^/A^2^)	Measured and fitted by parabolic equation
Temperature coefficient, *β*_1_	0.3981				
Temperature coefficient, *β*_2_	0.2336				
Temperature coefficient, *β*_3_	1.7220				

## Data Availability

The datasets generated during and/or analyzed during the current study are available from the corresponding author on reasonable request.
